# TMT-based quantitative proteomic analysis revealed that FBLN2 and NPR3 are involved in the early osteogenic differentiation of mesenchymal stem cells (MSCs)

**DOI:** 10.18632/aging.204931

**Published:** 2023-08-04

**Authors:** Jianyun Liu, Shan He, Baicheng Ma, Xingnuan Li, Yaqin Wang, Jianjun Xiong

**Affiliations:** 1 Jiangxi Provincial Key Laboratory of Systems Biomedicine, Jiujiang University, Jiujiang 332005, China; 2 Reproductive Medical Center, Renmin Hospital of Wuhan University, Wuhan 430060, China

**Keywords:** TMT-based proteomics, mesenchymal stem cell (MSC), differentiation, FBLN2, NPR3

## Abstract

The delicate equilibrium between osteoblast and adipocyte differentiation of MSCs is highly regulated. We screened for early-stage osteogenesis- or adipogenesis-based MSCs protein expression profiles using TMT-based quantitative proteomic analysis to identify novel participating molecules. Protein annotation, hierarchical clustering, functional stratification, and protein-protein association assessments were performed. Moreover, two upregulated proteins, namely, FBLN2 and NPR3, were validated to participate in the osteogenic differentiation process of MSCs. After that, we independently downregulated FBLN2 and NPR3 over seven days of osteogenic differentiation, and we performed quantitative proteomics analysis to determine how different proteins were regulated in knockdown vs. control cells. Based on gene ontology (GO) and network analyses, FBLN2 deficiency induced functional alterations associated with biological regulation and stimulus-response, whereas NPR3 deficiency induced functional alterations related to cellular and metabolic processes, and so on. These findings suggested that proteomics remains a useful method for an in-depth study of the MSCs differentiation process. This will assist in comprehensively evaluating its role in osteoporosis and provide additional approaches for identifying as-yet-unidentified effector molecules.

## INTRODUCTION

Mesenchymal stem cells (MSCs) possess strong self-renewal and directional differentiation abilities. Under various induction stimuli, MSCs can differentiate into osteoblasts, adipocytes, chondrocytes, etc., allowing it a wide range of biomedical engineering applications [1, 2]. Scientists are paying more attention to MSC involvement in osteoporotic research because such disorders are connected with an imbalance in MSC differentiation and osteoblast-osteoclast activity. MSC differentiation into adipocytes rather than osteoblasts is a common clinical feature in osteoporotic patients [3, 4]. Hence, MSCs are frequently used as a reliable *in vitro* induction model for exploring numerous modulatory factors and biomarkers during MSCs differentiation into osteoblasts/adipocytes [5, 6].

Numerous genes modulate MSCs differentiation into osteoblasts/adipocytes [7, 8]. Scientists have extensively examined the gene expression profile during MSCs differentiation in prior investigations at the genetic, transcription, post-transcription, translation, and post-translation levels [9–13]. Proteomics analysis provides a systematic approach to investigating changes in cell protein composition over time. It enables the extraction of relevant information and rules from high-throughput data investigations, as well as providing clues and a foundation for deciphering linked functional mechanisms [14]. In recent years, this technique has been extensively used in the field of MSCs research, particularly in the identification of MSCs phenotype [15], recognition of hallmark proteins during differentiation [16], comparative secretome analysis [17], and so on. The studies above provided extensive information on the underlying mechanism behind MSCs directional differentiation. However, there is a limitation to this process. Target protein detection depth by two-dimensional gel electrophoresis (2-DE) is limited in the early stages of proteomics application. Additionally, certain differentially regulated proteins during the differentiation of MSCs into osteoblasts and adipocytes remain poorly studied.

Therefore, we employed TMT (Tandem Mass Tags)-based quantitative proteomic analysis to assess the early MSC differentiation process in this study. The lineage commitment of MSC differentiation at specific stages, particularly at 0, 3, and 7 days after differentiation, was the focus of our effort to uncover various protein expression profiles. The osteogenesis/adipogenesis process was also the subject of proteome identification and comparative studies. We tentatively investigated the underlying functions of two differentially expressed (DE) proteins, FBLN2 and NPR3, using comparative proteomics analysis during MSCs differentiation. Our findings demonstrated a horizontal comparison mechanism and a vertical dynamic protein expression alteration. The results of the current study will significantly improve our knowledge of the role played by MSCs in osteoporosis and offer new information and methods for diagnosing and treating osteoporosis.

## MATERIALS AND METHODS

### MSCs culture and osteogenic/adipogenic induction

This work received ethical approval from Jiujiang University, Jiangxi, China. As reported previously, MSC extraction, identification, maintenance, and storage were conducted [18]. 6th generation MSCs were cultured for 14 days in MEM α with 10% FBS, 100 mM dexamethasone, 10 mM sodium glycerophosphate, and 50 ng/ml vitamin C to induce osteoblast differentiation, and osteogenesis was detected using Alizarin Red staining (ARS). Next, upon a 15 min stain elution using 10% cetylpyridinium chloride in 10 mM sodium phosphate solution, absorbance was read at 590 nm via a spectrophotometer. To induce adipogenesis, 6^th^ generation MSCs were cultured in minimum essential medium (MEM) α with 10% fetal bovine serum (FBS), 1.0 μM dexamethasone, 0.5 mM 3-isobutyl-1-methylxanthine, and 0.01 mg/ml insulin for 14 days. The adipogenic potential following adipogenic induction was confirmed through Oil Red O staining.

### Protein extraction and digestion

Cells were divided into five groups, namely, undifferentiated MSCs (MSC), osteogenic day 3 (OS3), osteogenic day 7 (OS7), adipogenic day 3 (AD3), and adipogenic day 7 (AD7). Following harvest, cells underwent sonication thrice in lysis buffer on ice via a high-intensity ultrasonic processor (Scientz) (8 M Urea, 1% Protease Inhibitor Cocktail), and debris removal was performed via a 10 min 12,000 g centrifugation at 4° C. A BCA kit was used to quantify the produced supernatant following kit instructions. To digest proteins, they were first reduced for 30 min at 56° C with 5 mM dithiothreitol and then alkylated for 15 min at RT in the dark with 11 mM iodoacetamide. The sample underwent dilution using 100 mM TEAB to a urea concentration <2M. Ultimately, trypsin was introduced at a 1:50 trypsin-to-protein mass ratio for an overnight (ON) initial digestion and a 4 h 1:100 trypsin-to-protein mass ratio secondary digestion.

### LC-MS/MS analyses and database screening

LC-MS/MS Analyses and Database Screening were carried out by the PTM Biolab (Hangzhou, China). In short, tryptic peptides were re-suspended in 0.1% formic acid (solvent A) before loading onto a laboratory-made reversed-phase analytical column. Our gradient consisted of an elevation from 6-23% solvent B (0.1% formic acid in 98% acetonitrile) for 26 min, 23-35% for 8 min, and 80% for 3 min before holding at 80% for the remaining 3 min. Subsequently, the peptides were exposed to an NSI source, with subsequent tandem mass spectrometry (MS/MS) in Q Exactive TM Plus (Thermo) attached to the UPLC. The parameters were adjusted: electrospray voltage to 2.0 kV, m/z scan range 350-1800 for a full scan, and intact peptides was identified in Orbitrap at 70,000 resolutions. Peptide selection for MS/MS used an NCE setting at 28, and fragment detection employed a 17,500 resolution in Orbitrap. Then, we used a data-dependent technique that rotated between 20 MS/MS scans with 15.0 s dynamic exclusions and a single MS scan. The parameter was set as follows: Fixed first mass: 100 m/z. The MS/MS data were processed via the MaxQuant search engine (v1.6.5.0). Tandem mass spectra were validated against the combined reverse decoy and human Uniprot databases.

Trypsin/P served as the cleavage enzyme, and a maximum of 4 missed cleavages were permitted. The following parameters were used: precursor ion mass tolerance: 20 ppm (preliminary screening) and 5 ppm (main screening), fragment ion mass tolerance: 0.02 Da, FDR: < 1%, minimum modified peptide score: >40. All experiments were conducted twice.

### Bioinformatics analysis

We used different databases or software to perform Gene Ontology (GO), protein domain, and the Kyoto Encyclopedia of Genes and Genomes database (KEGG). GO analyses were conducted via three major classifications: biological process (BP), cellular compartment (CC), and molecular function (MF). KEGG analyses involved the identification of critical networks related to the pre-identified DE proteins. To perform protein domain enrichment analysis, we used InterPro, a software application that categorizes protein sequences into families to determine crucial domains and locations. A p-value of <0.05 was adopted as the significant level in all analyses described above, and a two-tailed Fisher's exact test was employed to identify DE protein enrichment. Lastly, protein-protein associations were assessed using the STRING database (https://string-db.org/).

### Western blot (WB)

Following total protein isolation, equal quantities of proteins underwent separation on 10% SDS-PAGE before transfer to PVDF membrane (Bio-Rad, CA, USA), with subsequent ON blocking in 5% non-fat milk at 4° C and 2 h exposure to targeted antibodies at RT. The employed antibodies were as follows: anti-COL11A1 (ab166606, Abcam), anti-COMMD5 (67043-1, Proteintech), anti-SRPX2 (66266-1, Proteintech), anti-LRRC1 (ab127568, Abcam), anti-NPR3 (A8138, ABclonal), anti-APOL2 (25925-1, Proteintech), anti-GGT5 (A14374, ABclonal), anti-FBLN2 (sc-271843, Santa Cruz) and anti-β-actin (ab8227, Abcam). All antibodies mentioned above were used in dilution 1:1000, except for anti-β-actin, which was used in 1:2000 dilution. Corresponding secondary HRP-conjugated antibodies were employed in 1:10000 or 5000 dilutions, and protein visualization was done via an enhanced chemiluminescence system and quantification via Image Lab software.

### RNA interference

Per kit directions, MSCs were incorporated with FBLN2- or NPR3-targeted shRNA lentiviral particles (Genechem, Shanghai, China). The target sequences are summarized in Supplementary Table 1. To induce transduction, 5 × 10^5^ cells were plated in 25 cm^2^ in complete medium for an ON incubation, before a 12 h infection with 5 μL of shRNA in 5 g/mL polybrene media mixture, with subsequent refreshing of the old transduction medium to complete medium. Cell harvest was performed 3 days post-transduction for WB-based protein analysis or osteogenic/ adipogenic induction.

### Availability of data and materials

Data are available via ProteomeXchange with identifier PXD041404.

## RESULTS

### Quantitative analysis of protein expression during early differentiation of MSC towards osteogenesis/adipogenesis

We initially assessed the inducible effects of osteogenic/adipogenic induction media to investigate the *in vitro* model of osteogenesis/adipogenesis. The 6^th^ generation of MSCs underwent ARS and ORO staining after a 14-day stimulation with the appropriate medium, confirming effective MSC differentiation (Supplementary Figure 1).

**Figure 1:**
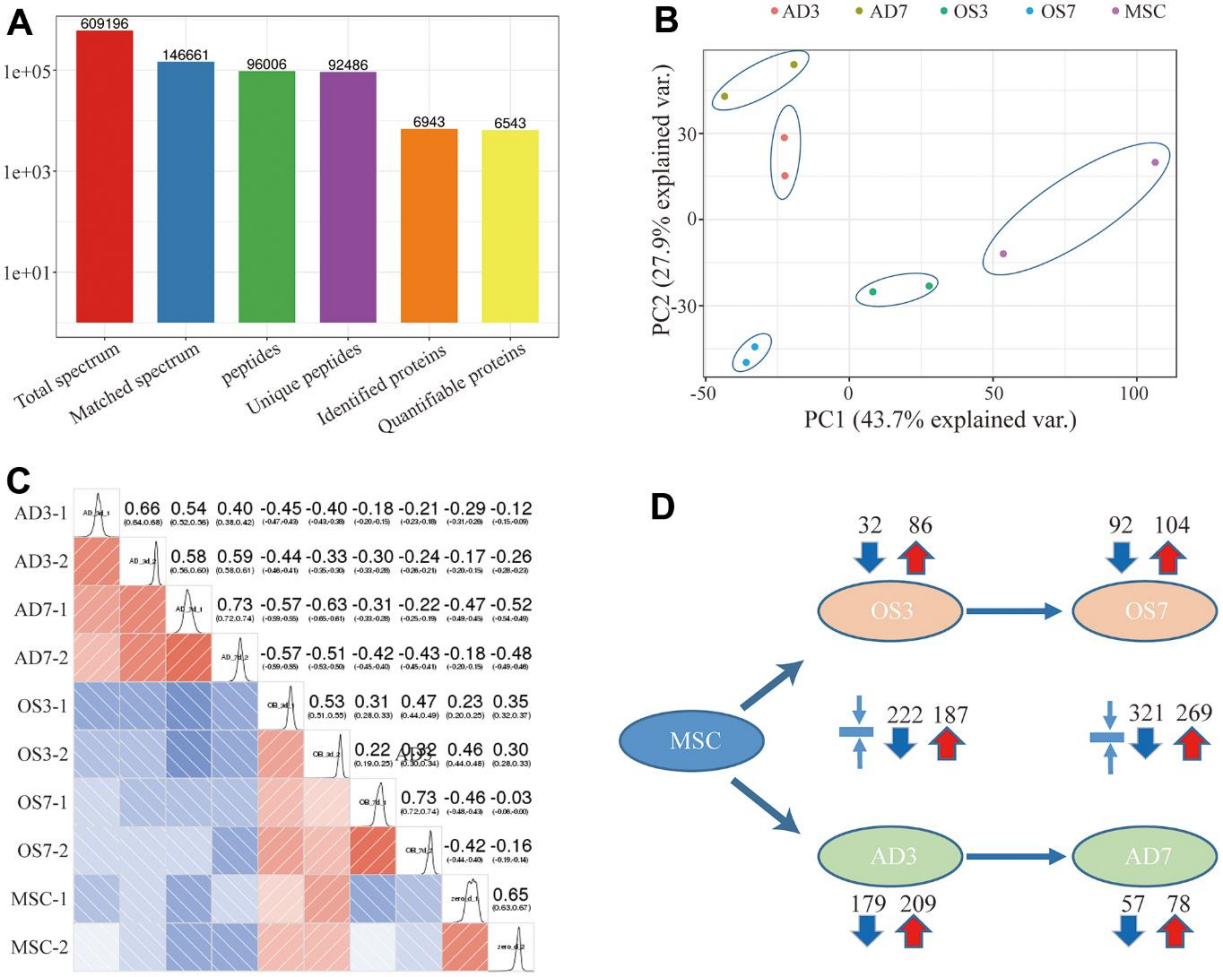
(**A**) Basic statistical figure depicting the MS data; (**B**) Two-dimensional scatter plot of the PCA (principal component analysis) profile of all samples, based on the protein quantification; (**C**) The heatmap, based on the Pearson correlation coefficient, shows all examined proteins between individual pairings of samples. (**D**) Quantities of highly- and scarcely-expressed proteins, as evidenced by proteomics.

Using TMT-based quantification, we identified 609196 secondary spectral proteins. Upon comparison of this data with the theoretical protein data, the number of available spectra was 146661, and the spectral utilization rate was about 24%. In all, 96006 peptides were screened via spectral analysis, among which 92486 specific peptide segments were identified. Moreover, out of the 6943 proteins screened in this investigation, 6543 provided quantitative information (Figure 1A). The samples demonstrated satisfactory reproducibility based on our principal component (PCA) and Pearson correlation coefficient analyses (Figure 1B, 1C). Significance was considered when the inter-group fold change was <0.67 or >1.5, and the *t*-test *p*-value was < 0.05. Relative to the MSCs, 86 proteins were highly expressed, and 32 were scarcely expressed in the OS3 group; 104 proteins were highly expressed, and 92 were scarcely expressed in the OS7 group; 209 proteins were highly expressed, and 179 were scarcely expressed in the AD3 group; and 78 proteins were highly expressed, and 57 were scarcely expressed in the AD7 group. In addition, we performed a cross-range comparison between the OS3 and AD3 groups or the OS7 and AD7 groups. In the OS3 group, compared to the AD3 group, we found 187 highly expressed proteins and 222 proteins with low expression. In addition, 269 proteins were expressed at high levels and 321 at low levels in the OS7 group compared to AD7 (Figure 1D).

On the 3^rd^ and 7^th^ days of osteogenesis, 38 proteins were significantly increased (over 1.5 folds), whereas, on the 3^rd^ and 7^th^ days of adipogenesis, 86 proteins were significantly increased (over 1.5 folds). These highly expressed proteins have potential research value and are detailed in Supplementary Table 2A, 2B.

### Bioinformatics analysis the differentially expressed proteins

**Figure 2:**
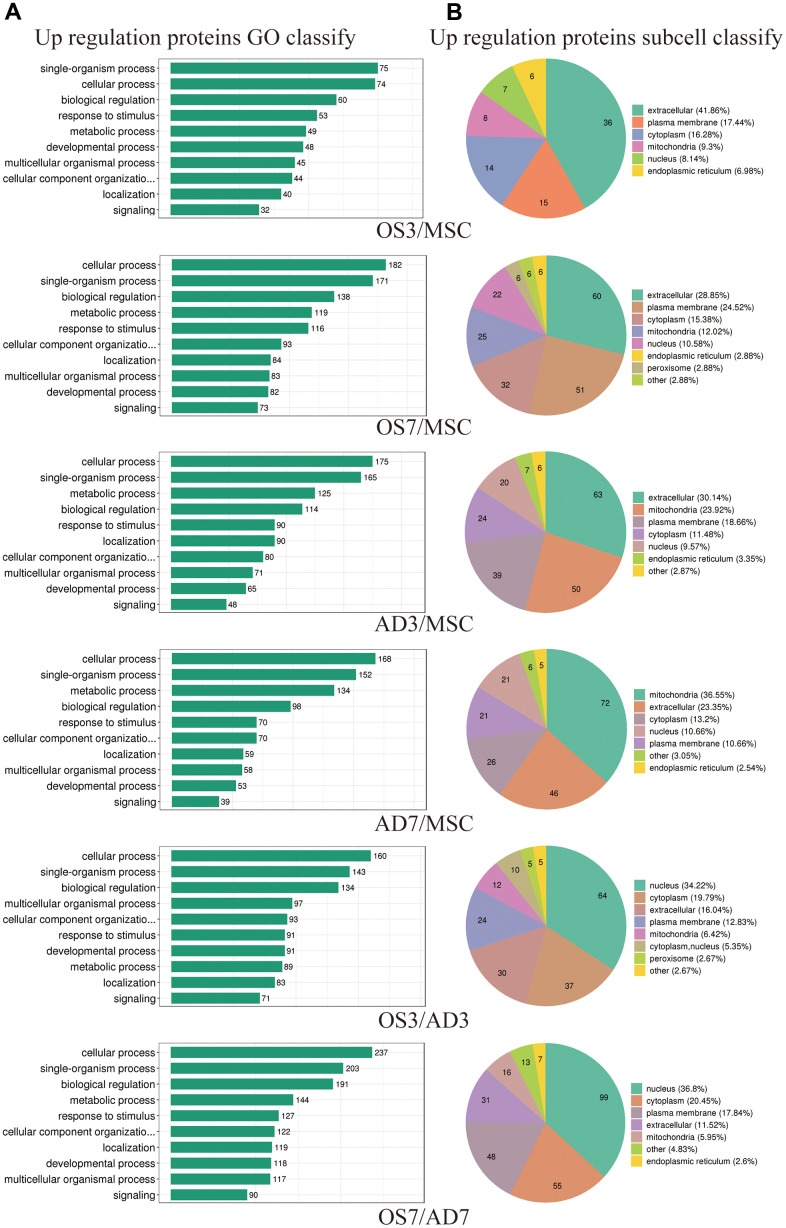
**Functional annotations of differentially regulated proteins.** (**A**) Statistical distribution of highly expressed proteins based on the GO secondary classification (Biological Process). (**B**) Classification of highly expressed protein subcellular structure.

We mainly focused on DE proteins that showed substantial overexpression according to bioinformatics analysis. Considering BP, the GO stratification of highly expressed proteins between each comparison group was largely similar and comprised physiological regulation, stimulus-response, cellular organization, and cellular, single-organism, and metabolic activities (Figure 2A). However, the subcellular location of highly expressed proteins exhibited considerably more variation between these comparison groups. For instance, (1) between the OS3 and MSC groups, there were 36 elevated proteins localized within the extracellular space, and between the OS7 and MSC groups, the number increased to 60. (2) between the AD3 and MSC groups, there were 50 enhanced proteins localized within the mitochondria, and between the OS7 and MSC groups, the number increased to 72. Lastly, (3) between the OS3 and AD3 or between the OS7 and AD7 groups, the maximum enhanced protein proportions were localized within the nucleus (Figure 2B).

**Figure 3:**
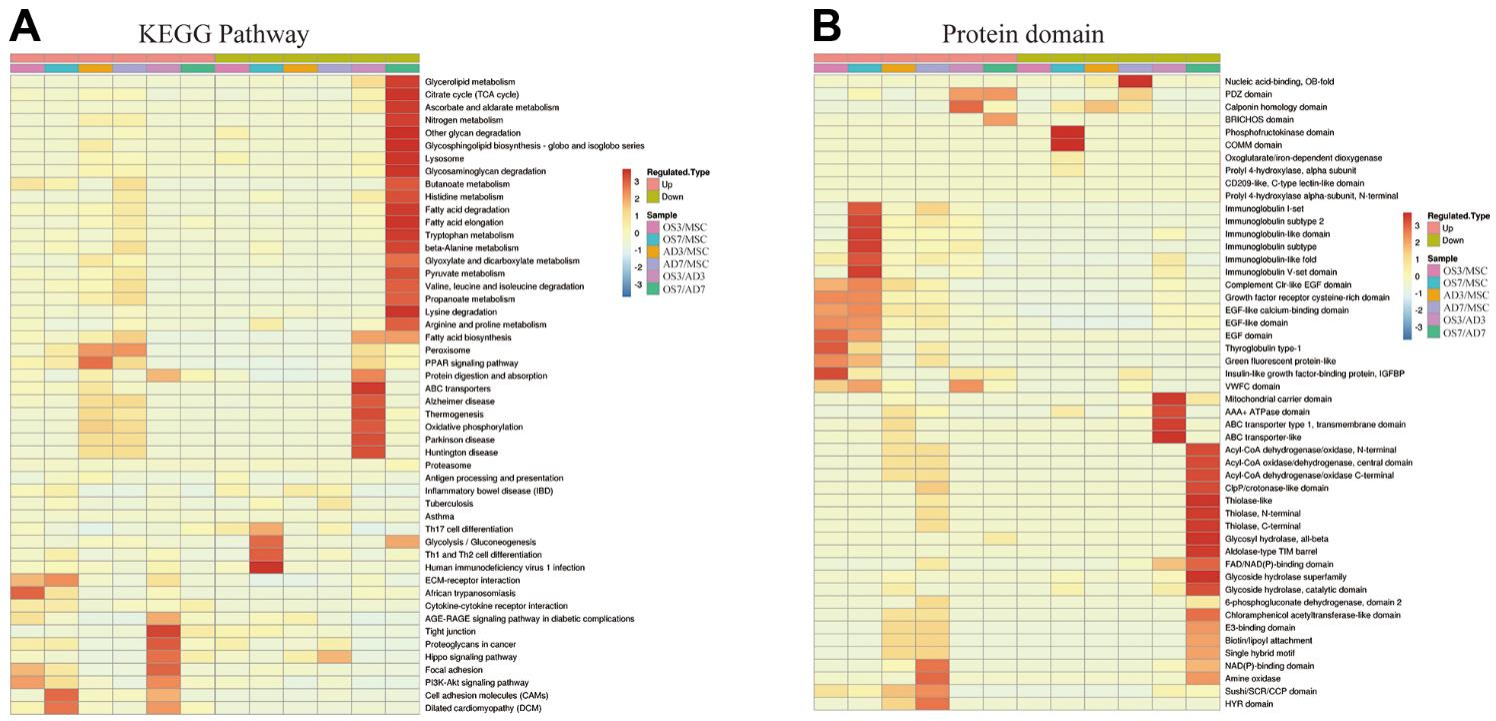
**Heat map cluster analysis.** (**A**) Based on the KEGG pathway cluster analysis, (**B**) Based on the protein domain cluster analysis.

The KEGG pathway enrichment-based cluster analysis revealed that, in the OS3 group, the highly expressed proteins were enriched in the ECM receiver interaction and focal adhesion pathway. In the OS7 group, they were enriched in the ECM receiver interaction and cell adhesion motors. In the AD3 and AD7 groups, the PPAR axis and peroxide were the primary clustering pathways. In addition, the KEGG clusters of DE proteins were substantially different between the osteogenic and adipogenic comparison (OS/AD). The highly expressed proteins in osteogenesis were clustered in the tight junction, focal adhesion, and cell adhesion molecules. In contrast, the highly expressed proteins in adipogenesis were clustered primarily in the fatty acid and glycolide metabolism networks, thus demonstrating distinct protein functions during osteogenic and adipogenic differentiation processes (Figure 3A).

The highly expressed proteins in the OS3 group were clustered in the insulin-like growth factor-binding protein and the EGF domains. In contrast, according to our protein domain clustering analysis, the highly expressed proteins in the OS7 group were clustered in the immunoglobulin-related domains. Highly expressed proteins in the AD3 or AD7 group were clustered in the NAD(P)-binding domain, amine oxidase domain, and so on. Between the adipogenesis and osteogenesis processes, the highly expressed proteins were clustered in numerous domains, such as the mitochondrial carrier domain, acyl-CoA-related domain, Thiolase domain, etc. (Figure 3B).

We determined the top 50 proteins in each comparison group with the highest number of direct associations to plot the protein association axis and better understand the relationships between DE proteins. The corresponding results are presented in Supplementary Figure 2.

### Expression validation

**Figure 4:**
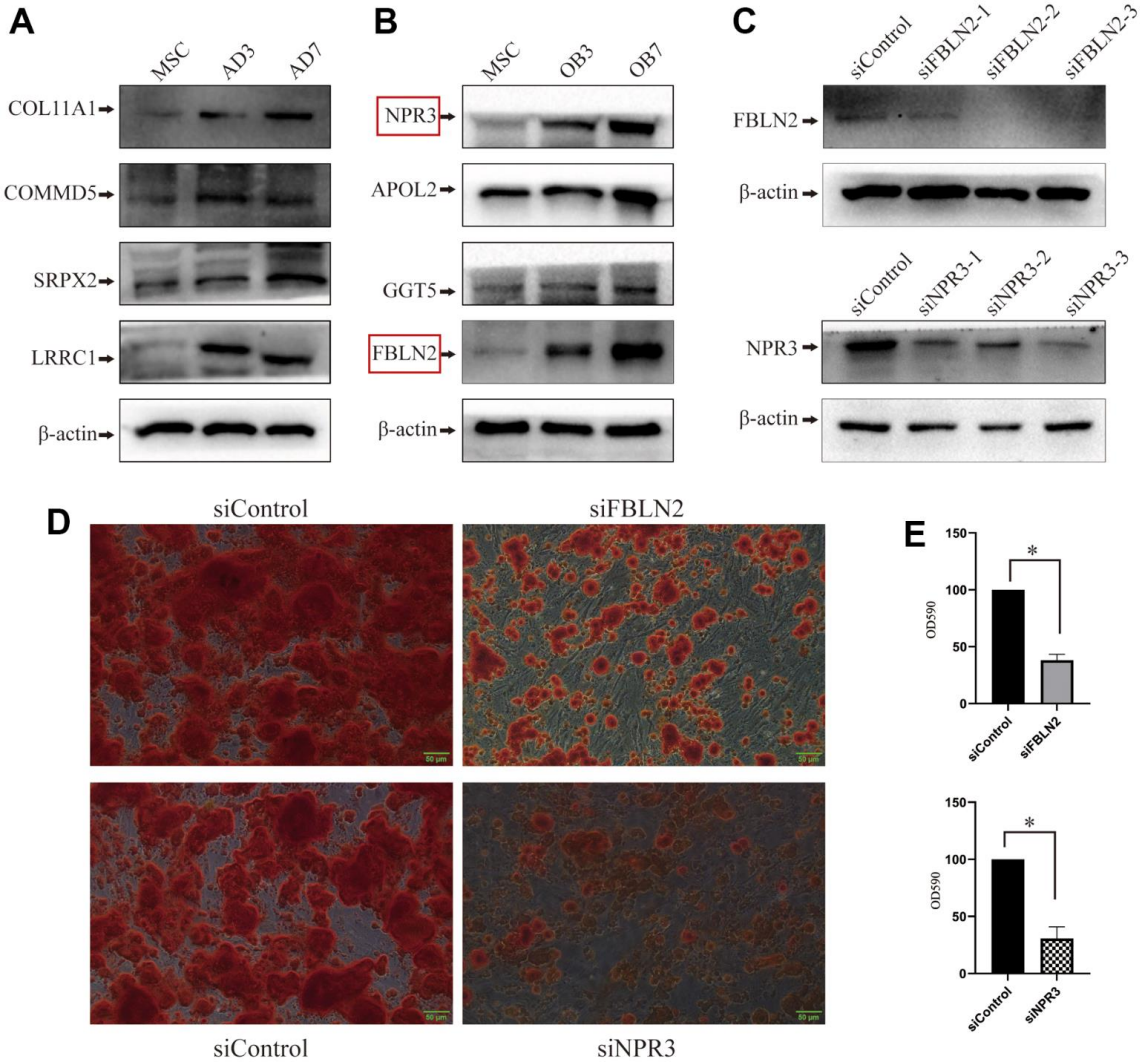
**Validation of proteomic sequencing.** (**A**) In all, 4 adipogenic differentiation-related proteins were validated by western blot (WB); (**B**) 4 osteogenic differentiation-related proteins were validated by western blot (the protein marked in the red box will undergo further examination); (**C**) Lentivirus-mediated shRNA delivery for the effective knockdown of FBLN2 or NPR3; (**D**) ARS displayed a decrease in the osteogenic ability of MSCs induced by FBLN2 or NPR3 deficiency; (**E**) Quantitative analysis of alizarin red staining (ARS) following knockdown of FBLN2 or NPR3.

We selected random 4 proteins (NPR3, APOL2, GGT5, and FBLN2) that showed substantially high expression during osteogenic differentiation and 4 proteins (COL11A1, COMMD5, SRPX2, and LRRC1) that showed significantly high expression during adipogenic differentiation for WB-based verification. Based on our analysis, the expression profile of the proteins, as mentioned above, during MSCs differentiation was consistent with data from proteomics-seq, thereby suggesting the accuracy of the sequencing data (Figure 4A, 4B).

### FBLN2 and NPR3 participate in the osteogenic differentiation of MSC

According to the proteomics-seq, FBLN2 and NPR3 were significant because they had previously received little attention when studying osteogenic differentiation. Using lentivirus-mediated shRNA delivery, we successfully and independently knocked down FBLN2 and NPR3 in these experiments (Figure 4C). FBLN2 or NPR3 deficiency did not influence MSCs proliferation within 7 days post-infection (Data not shown). However, following 48 h of incorporation, MSCs were exposed to osteogenic inducer for 14 days. ARS revealed that FBLN2 or NPR3 knockdown partially suppressed extracellular calcification during osteogenesis (Figure 4D). Using quantitative analysis of ARS, we demonstrated that FBLN2 deficiency reduced ARS by approximately 60% and NPR3 deficiency by 65% (Figure 4E).

### Functions of FBLN2 or NPR3, as evidenced by quantitative proteomics analysis

**Figure 5:**
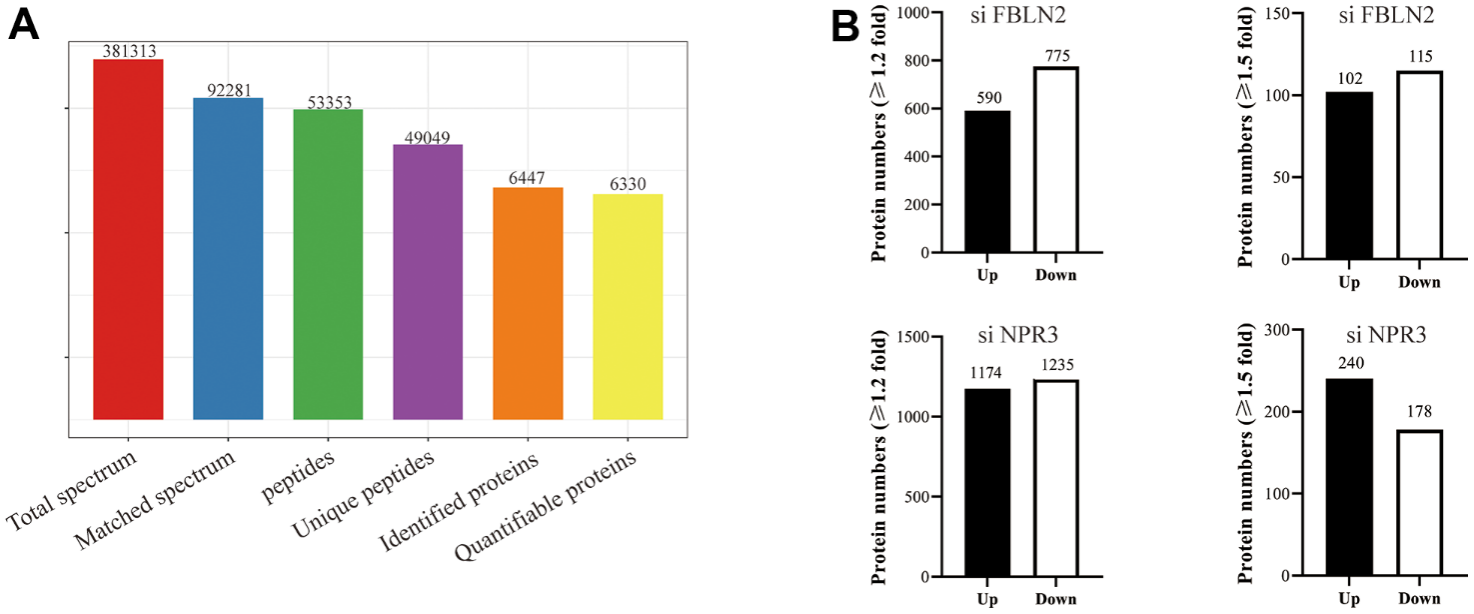
(**A**) Basic MS data in the statistical form; (**B**) Quantities of different protein expressions from the proteomics analyses on FBLN2 or NPR3 knockdown.

We used quantitative proteomics to evaluate the DE proteins on day 7 of MSC osteogenic differentiation after FBLN2 or NPR3 deficiency to investigate the underlying processes behind the FBLN2- and NPR3-mediated impact on MSC osteogenic differentiation. Overall, 381313 peptides were screened by spectral analysis, among which specific peptide segments were observed in 53353 proteins. In all, 6447 proteins were screened in this investigation, among which 6330 proteins provided quantitative information (Figure 5A). Compared to the parental control cells, FBLN2 knockdown resulted in 590 highly expressed proteins, 775 scarcely expressed proteins (by 1.2-fold), 102 highly expressed proteins, and 115 scarcely expressed proteins (by 1.5-fold). In the NPR3 knockdown cells, 1174 and 1235 proteins were markedly elevated and diminished (by 1.2-fold), respectively, or 240 and 178 proteins were strongly elevated and decreased (by 1.5-fold), respectively, relative to the parental control cells (Figure 5B).

**Figure 6:**
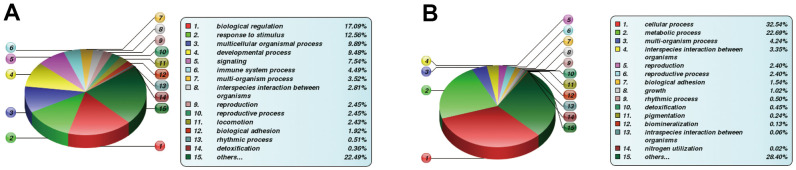
**Differentially expressed (DE) protein stratification using GO secondary analysis.** (**A**) Statistical Profile of DE proteins in GO secondary stratification (Biological Process) between FBLN2 deficient and control cells; (**B**) Statistical profile of DE proteins in GO secondary stratification (Biological Process, BP) between NPR3 deficient and control cells.

**Figure 7:**
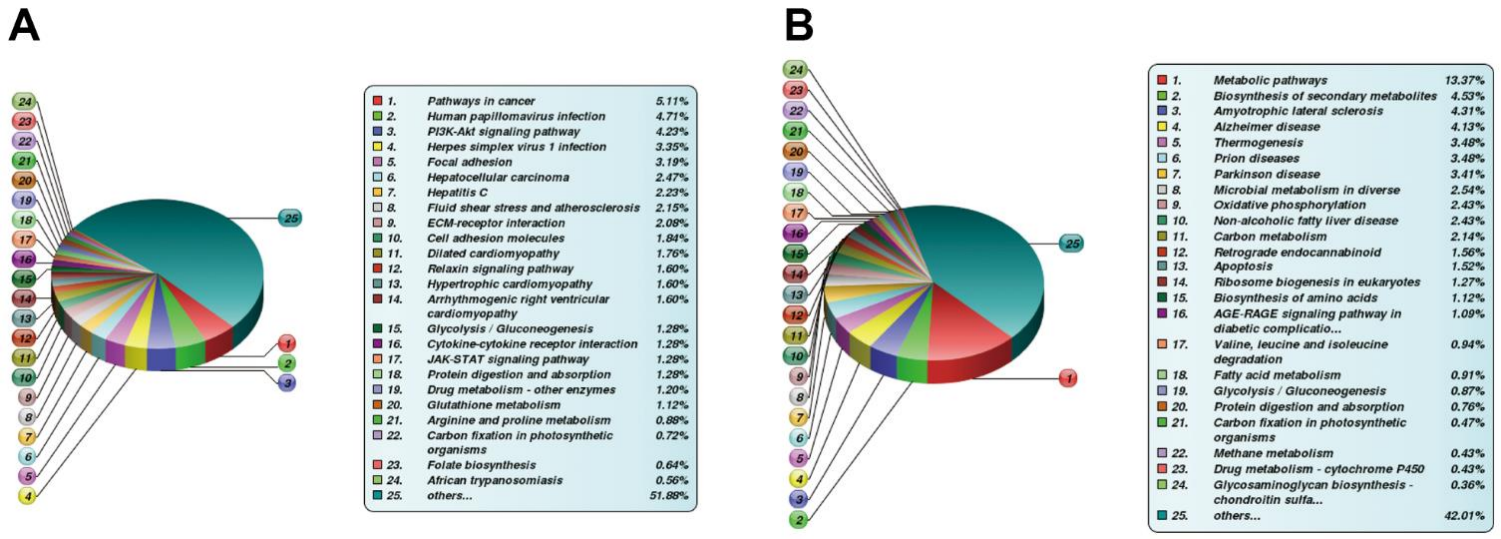
**Differentially expressed (DE) proteins are enriched via the KEGG pathway.** (**A**) KEGG analysis between FBLN2 deficient and control cells; (**B**) KEGG analysis between NPR3 deficient and control cells.

The DE proteins were then subjected to bioinformatics analysis. Concerning the BP category, DE proteins were primarily enriched in physiological modulation and stimulus-response between the FBLN2 deficient and control groups at OS7. In the NPR3 deficient versus control group, DE proteins were increased in the cellular and metabolic processes (Figure 6). Based on the KEGG network enrichment analysis, DE proteins between the FBLN2 deficient and control groups were relatively scattered, including cancer-associated pathways, PI3K-AKT axis, focal adhesion, etc. By comparison, over 10% of DE proteins between the NPR3 deficient and control groups were clustered in the metabolic pathway (Figure 7).

**Figure 8:**
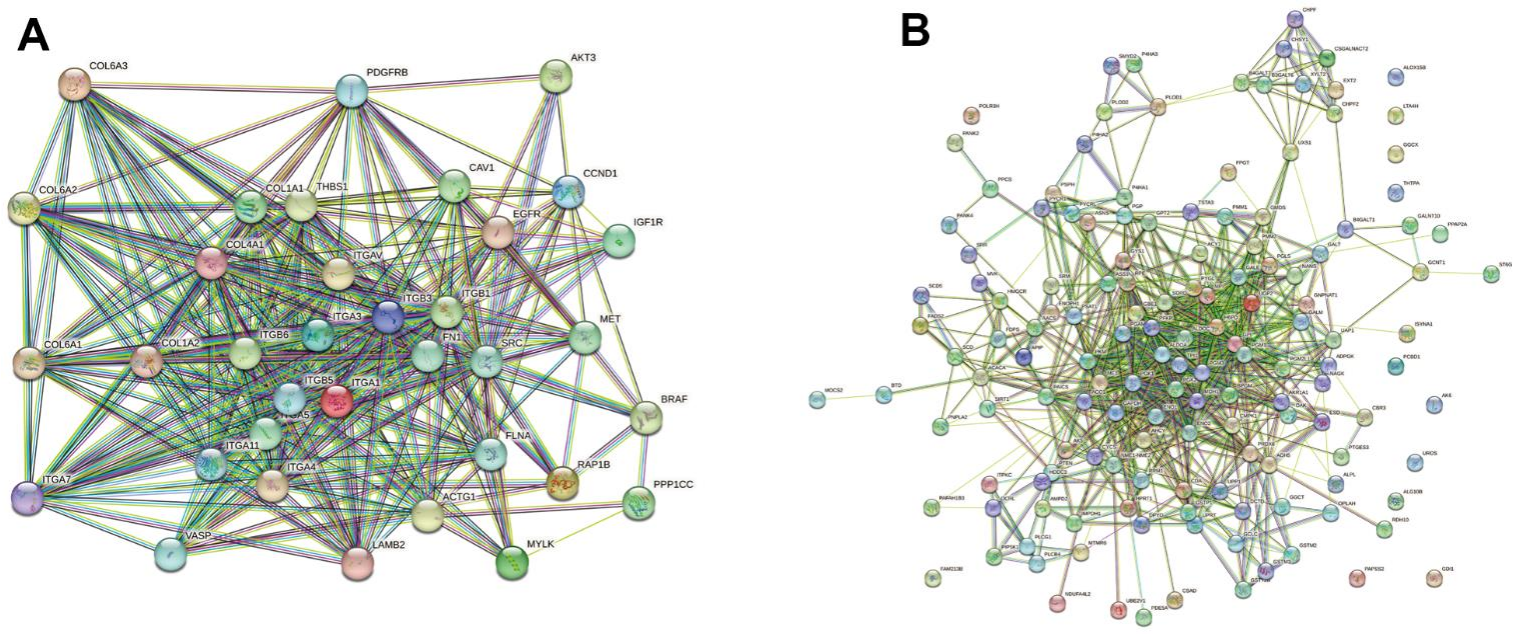
**Network diagram of differentially expressed (DE) protein association.** (**A**) In the FBLN2 knockdown cells, we performed PPI analysis on 35 scarcely expressed proteins associated with the focal adhesion pathway; (**B**) In the NPR3 knockdown cells, we performed PPI analysis on 146 scarcely expressed proteins related to the metabolic pathway.

The PPI networks of DE proteins were assessed using the STRING network. Among DE proteins between the FBLN2 deficient and control groups, we selected 35 scarcely expressed proteins associated with the focal adhesion pathway for PPI analysis. The ITG and collagen protein families, in particular, were employed to construct the major PPI complex network (Figure 8A). Among DE proteins between the NPR3 deficient and control groups, we selected 146 scarcely expressed proteins associated with the metabolic pathway for PPI analysis. The associations between DE proteins were observed to be more dispersed and complex (Figure 8B).

## DISCUSSION

An intricate balance exists in differentiating MSCs to osteoblasts and adipocytes, regulated by a precise expression of multiple genes in time and space to guarantee appropriate MSC differentiation [19]. Proteomics, based on 2D gel electrophoresis, is a long- standing protein detection method during MSC differentiation. Recently, highly efficient TMT labeling technology was introduced into proteomics, which enhanced the detection of relative protein expression [20]. In the present study, we employed TMT-labeled technologies to identify and analyze DE proteins relevant to the osteogenic/adipogenic differentiation process of MSCs. Our findings will probably increase our knowledge of the critical protein molecules that support the delicate balance of MSC development and provide us with a more thorough understanding of the gene expression pattern during early MSC differentiation.

Our proteomics research found more DE genes than earlier proteomics based on 2D gel electrophoresis. The number of DE proteins was higher in each sequencing result than in the combined study. Although we acquired more extensive sequencing data, it was intriguing that the DE proteins were incompatible with earlier researchers' findings. For example, during MSC osteogenesis, Ai Xia Zhang’s [21] identified 52 DE proteins with 2.0-fold changes in expression during osteogenic differentiation. However, only a few proteins of those proteins, such as annexin A2, FKBP5, and HMG-1, were on our list of DE proteins, and other reported DE proteins, such as Efhc2, nascent polypeptide associated (NACA), RBBP9, and OSBPL7 were absent from our list. Our study of adipogenic differentiation yielded similar results. Hyun Kyung Lee discovered 8 highly expressed proteins with 5.0-fold changes in MSC differentiation to adipocytes [22]. Among the reported 8 proteins, only OSBP-related protein 3 was detected in our sequencing list. We speculated that the possible reasons for diversity in our sequencing results were as follows: 1) cellular protein expressions are typically in dynamic change at all times, 2) the number of cellular passages may have affected protein expression, and 3) the purity of MSCs impacted the protein expression. In the effort to overcome the obstacles mentioned above, it is essential to increase the number of repetitions to improve the reliability of the sequencing results.

Under the treatment of osteogenic/adipogenic inducers, different gene expression leads to MSC differentiation in different directions. Using cross-range comparative analysis of the protein expression profile at the same time point during osteogenic/adipogenic differentiation, we observed that certain proteins displayed the same expression trend during both osteogenic and adipogenic differentiation, with 26 upregulated proteins on the third day of differentiation and 43 upregulated proteins on the seventh day of differentiation, respectively. As a result, these proteins may be required for differentiation but not engaged in differentiation selection. NID1 was one such protein, a member of the nidogen family of basement membrane glycoproteins. This protein interacts with several other basement membrane components to contribute to the interaction between cells and the extracellular matrix [23]. In addition, several proteins exhibited different degrees of expression between osteogenic and adipogenic differentiation. For example, COL1A1 displayed the largest multiple differences on the third day of differentiation between osteogenesis and adipogenesis. It is well known that COL1A1 is a marker of osteogenesis and has a very close relationship with bone formation [24]. Given the seesaw effect of osteogenic/lipogenic differentiation, we must consider these proteins with considerable differential expression between the two processes. Because of their unique indirect physiological activities associated with MSC differentiation, we selected FBLN2 and NPR3 proteins as candidate differentiation regulating proteins/markers for further validation investigation in the context of osteoporotic pathogenesis. The FBLN2 gene encodes an extracellular matrix (ECM) protein, which binds multiple extracellular ligands and calcium [25], and it has a wide range of physiological functions. Some studies revealed a strong relationship between FBLN2 and the occurrence and progression of tumors [26, 27]. Other studies reported that FBLN2 is likely associated with cardiovascular disease [28, 29]. Our interest in FBLN2 stemmed from its strong expression in osteoblasts [30]. We showed that FBLN2 knockdown severely interfered with MSCs’ ability to differentiate into osteoblasts. We additionally found through proteomics analysis that some molecules from the PI3K and focal pathways may be downstream targets of FBLN2, which requires further investigation.

Another complicated issue is the activation of FBLN2 during osteogenic differentiation. A previous study found that glucocorticoids reduce FBLN2 expression in bone marrow stromal cells [31], which contradicts its increased expression during osteoblastic differentiation if the osteogenic inducer contains dexamethasone. A previous report revealed that FBLN2, a downstream target molecule of TGF-β, is activated by the TGF-β pathway in liver myofibroblasts [31]. However, whether the TGF signaling pathway activates FBLN2 during the osteogenesis of MSCs is unknown. NPR3 is another DE protein examined in this study. The NPR3 gene is located on the human chromosome 5p13.3 and encodes one of three natriuretic peptide receptors [32]. The signaling pathway consists of a natriuretic peptide and its receptor, and it is involved in modulating blood volume and pressure, pulmonary hypertension, cardiac function, and other physiological activities [33]. Excessive natriuretic peptide production results in bone overgrowth and skeletal abnormalities in young children, particularly affecting their height, vertebrae, and finger length, thereby indicating a strong role in the modulation of endochondral ossification and bone development [34]. Mice with NPR3 mutation exhibit enhanced natriuretic peptide activity and present with a bone overgrowth phenotype, which results in a slender body and hunchback, similar to the phenotype produced by natriuretic peptide overexpression [35]. During MSC osteogenic differentiation, NPR3, RUNX2, DLX5, and other osteogenic genes were markedly enhanced, which may be the result of dexamethasone stimulation [36], however, the role of NPR3 in modulating osteoblastic differentiation remains unclear. For the first time, our findings revealed that NPR3 loss may impair MSC osteogenic development. It is important to note that during MSC osteogenic development, the proteins changed by NPR3 lack were predominantly enriched in the metabolic pathway, indicating that NPR3 deficiency will open up a new avenue of investigation into the molecular mechanism underlying NPR3 activity.

Although we attempted to explore the underlying molecular mechanisms regulating the early stage of MSCs differentiation using TMT-labeled proteomics, this study has certain deficiencies. Firstly, more detailed or extended time points are necessary for sequencing analysis of MSC differentiation. Secondly, there was no systematic detection of secretory proteins during differentiation. Additionally, only at the cellular level—not at the animal level—our verified FBLN2 and NPR3 proteins were. Future investigations will address the deficiencies mentioned above.

## CONCLUSIONS

In the current study, we employed TMT-based proteomics to analyze the expression profile of MSC at the early stage of osteogenic/adipogenic differentiation and identified several new differentially expressed proteins. Our sequencing results confirmed the highly expressed FBLN2 and NPR3 participate in the early osteogenic differentiation process. Collectively, these data will provide a new basis for further research into the molecular mechanism of diseases such as osteoporosis or adipogenesis.

## Supplementary Material

Supplementary Figures

Supplementary Tables 1 and 2A

Supplementary Table 2B
