# Supplementary material for: TMT-based quantitative proteomic analysis revealed that FBLN2 and NPR3 are involved in the early osteogenic differentiation of mesenchymal stem cells (MSCs)
Source: Aging (Albany NY). 2023 Aug 4;15(15):7637–54. doi: 10.18632/aging.204931 (PMC10457061; doi:10.18632/aging.204931)
Supplement: Supplementary Figures [file aging-15-204931-s001.pdf]

SUPPLEMENTARY FIGURES

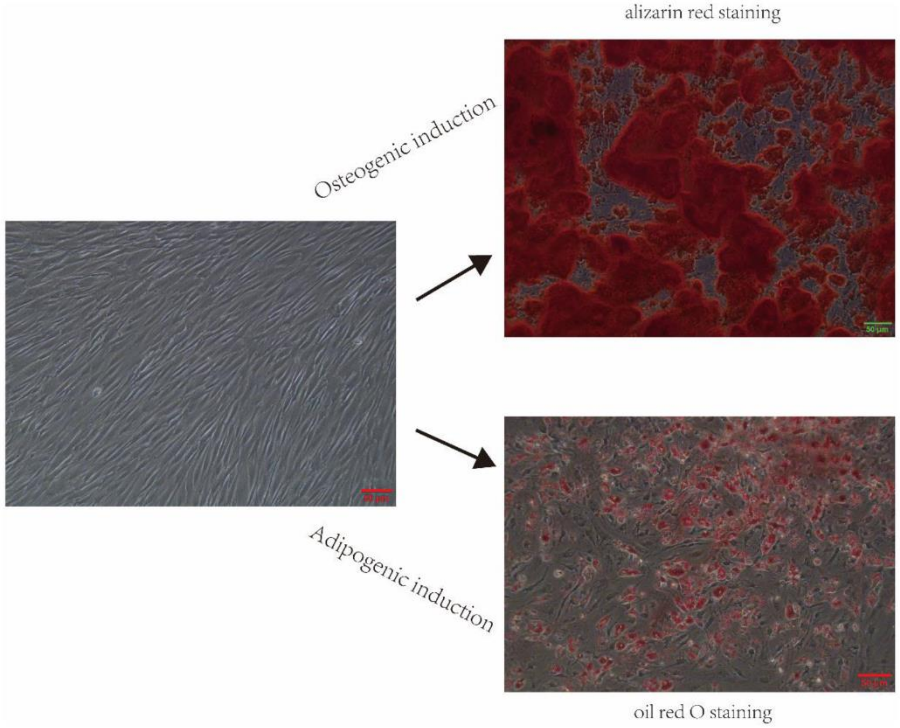

Supplementary Figure 1. Following a 14-day stimulation with osteogenesis/adipogenesis media, the 6th generations MSCs underwent ARS and ORO staining.

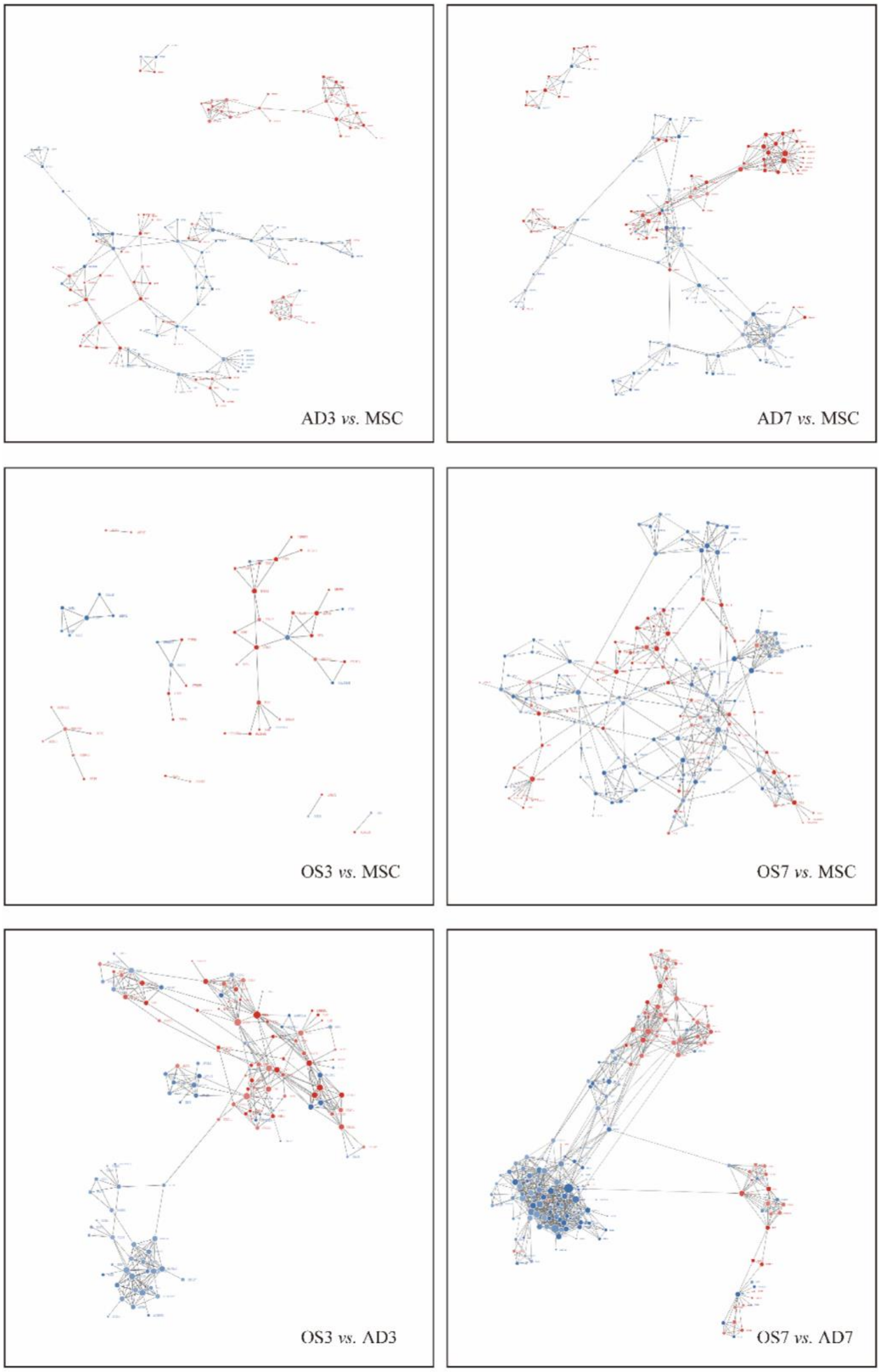

Supplementary Figure 2. Interaction network of differentially expressed proteins. The circle in the figure represents the differential expression of proteins, and different colors represent the differential expression of proteins (blue is down-regulated protein, red is up-regulated protein). The size of the circle represents the number of differential proteins and their interacting proteins.
