# Supplementary material for: TMT-based quantitative proteomic analysis revealed that FBLN2 and NPR3 are involved in the early osteogenic differentiation of mesenchymal stem cells (MSCs)
Source: Aging (Albany NY). 2023 Aug 4;15(15):7637–54. doi: 10.18632/aging.204931 (PMC10457061; doi:10.18632/aging.204931)
Supplement: Supplementary Tables 1 and 2A [file aging-15-204931-s002.pdf]

SUPPLEMENTARY TABLES

Supplementary Table 1. The target sequences designed for RNA interference.

NO.	Target Seq	GC%
FBLN2-RNAi-1	gcCTGCACTGAAGTCAGAATT	42.11%
FBLN2-RNAi-2	ccCAAAGTTGACATTCCATTT	31.58%
FBLN2-RNAi-3	ccTGAACATCATCAAGGGCAA	42.11%
NPR3-RNAi-1	ccGAATTGTAGAGCATACAAA	31.58%
NPR3-RNAi-2	ccAGGAGGTTATTGGTGATTA	36.84%
NPR3-RNAi-3	ccTGAGATTCTTTAAGGAGAT	31.58%
Negative control	TTCTCCGAACGTGTCACGT	52.63%

Supplementary Table 2A. Proteins were markedly enhanced (over 1.5 folds) on the 3rd and 7th days of osteogenesis.

Gene name	Protein accession	Protein description	OS3/MSC ratio	OS7/MSC ratio
COMP	P49747	Cartilage oligomeric matrix protein OS=Homo sapiens OX=9606 GN=COMP PE=1 SV=2	5.3175	4.416
NPR3	P17342	Atrial natriuretic peptide receptor 3 OS=Homo sapiens OX=9606 GN=NPR3 PE=1 SV=2	3.2535	3.6915
APOD	P05090	Apolipoprotein D OS=Homo sapiens OX=9606 GN=APOD PE=1 SV=1	2.5845	9.7255
ITGA1	P56199	Integrin alpha-1 OS=Homo sapiens OX=9606 GN=ITGA1 PE=1 SV=2	2.5395	3.547
GGT5	P36269	Glutathione hydrolase 5 proenzyme OS=Homo sapiens OX=9606 GN=GGT5 PE=1 SV=2	2.387	4.002
TMEM173	Q86WV6	Stimulator of interferon genes protein OS=Homo sapiens OX=9606 GN=TMEM173 PE=1 SV=1	2.351	2.352
CTDSPL	O15194	CTD small phosphatase-like protein OS=Homo sapiens OX=9606 GN=CTDSPL PE=1 SV=2	2.24	2.2555
IFNGR1	P15260	Interferon gamma receptor 1 OS=Homo sapiens OX=9606 GN=IFNGR1 PE=1 SV=1	2.201	5.372
HSPG2	P98160	Basement membrane-specific heparan sulfate proteoglycan core protein OS=Homo sapiens OX=9606 GN=HSPG2 PE=1 SV=4	2.1925	3.4065
APOL2	Q9BQE5	Apolipoprotein L2 OS=Homo sapiens OX=9606 GN=APOL2 PE=1 SV=1	2.156	3.6015
PCK2	Q16822	Phosphoenolpyruvate carboxykinase [GTP], mitochondrial OS=Homo sapiens OX=9606 GN=PCK2 PE=1 SV=4	2.1095	4.1915
CYR61	O00622	Protein CYR61 OS=Homo sapiens OX=9606 GN=CYR61 PE=1 SV=1	2.064	2.371
PLXNB1	O43157	Plexin-B1 OS=Homo sapiens OX=9606 GN=PLXNB1 PE=1 SV=3	2.046	2.7105
RECK	O95980	Reversion-inducing cysteine-rich protein with Kazal motifs OS=Homo sapiens OX=9606 GN=RECK PE=1 SV=1	2.0435	2.468
METTL7A	Q9H8H3	Methyltransferase-like protein 7A OS=Homo sapiens OX=9606 GN=METTL7A PE=1 SV=1	2.0185	3.0715
NID1	P14543	Nidogen-1 OS=Homo sapiens OX=9606 GN=NID1 PE=1 SV=3	2.0095	2.6885
DHCR24	Q15392	Delta (24)-sterol reductase OS=Homo sapiens OX=9606 GN=DHCR24 PE=1 SV=2	1.9475	2.8385
MOCS1	Q9NZB8	Molybdenum cofactor biosynthesis protein 1 OS=Homo sapiens OX=9606 GN=MOCS1 PE=1 SV=3	1.9465	1.995
FN1	P02751	Fibronectin OS=Homo sapiens OX=9606 GN=FN1 PE=1 SV=4	1.938	3.029
TMEM120B	A0PK00	Transmembrane protein 120B OS=Homo sapiens OX=9606 GN=TMEM120B PE=1 SV=1	1.8655	1.909
FAS	P25445	Tumor necrosis factor receptor superfamily member 6 OS=Homo sapiens OX=9606 GN=FAS PE=1 SV=1	1.861	3.008
STOM	P27105	Erythrocyte band 7 integral membrane protein OS=Homo sapiens OX=9606 GN=STOM PE=1 SV=3	1.8585	3.24

ADAMTSL4	Q6UY14	ADAMTS-like protein 4 OS=Homo sapiens OX=9606 GN=ADAMTSL4 PE=1 SV=2	1.8525	3.7745
MYO1C	O00159	Unconventional myosin-Ic OS=Homo sapiens OX=9606 GN=MYO1C PE=1 SV=4	1.7915	1.9125
GRAMD2B	Q96HH9	GRAM domain-containing protein 2B OS=Homo sapiens OX=9606 GN=GRAMD2B PE=1 SV=1	1.783	2.167
CALCOCO2	Q13137	Calcium-binding and coiled-coil domain-containing protein 2 OS=Homo sapiens OX=9606 GN=CALCOCO2 PE=1 SV=1	1.779	1.935
NID2	Q14112	Nidogen-2 OS=Homo sapiens OX=9606 GN=NID2 PE=1 SV=3	1.7685	2.843
THSD4	Q6ZMP0	Thrombospondin type-1 domain-containing protein 4 OS=Homo sapiens OX=9606 GN=THSD4 PE=2 SV=2	1.7535	2.8155
FBLN2	P98095	Fibulin-2 OS=Homo sapiens OX=9606 GN=FBLN2 PE=1 SV=2	1.702	2.6035
HP	P00738	Haptoglobin OS=Homo sapiens OX=9606 GN=HP PE=1 SV=1	1.6835	2.093
EFEMP2	O95967	EGF-containing fibulin-like extracellular matrix protein 2 OS=Homo sapiens OX=9606 GN=EFEMP2 PE=1 SV=3	1.6125	2.7705
SCUBE3	Q8IX30	Signal peptide, CUB and EGF-like domain-containing protein 3 OS=Homo sapiens OX=9606 GN=SCUBE3 PE=1 SV=1	1.6	1.68
BOK	Q9UMX3	Bcl-2-related ovarian killer protein OS=Homo sapiens OX=9606 GN=BOK PE=1 SV=1	1.598	1.815
PLAC9	Q5JTB6	Placenta-specific protein 9 OS=Homo sapiens OX=9606 GN=PLAC9 PE=1 SV=1	1.5565	3.1535
SVEP1	Q4LDE5	Sushi, von Willebrand factor type A, EGF and pentraxin domain-containing protein 1 OS=Homo sapiens OX=9606 GN=SVEP1 PE=1 SV=3	1.5275	1.515
ACSL1	P33121	Long-chain-fatty-acid--CoA ligase 1 OS=Homo sapiens OX=9606 GN=ACSL1 PE=1 SV=1	1.524	2.218
STBD1	O95210	Starch-binding domain-containing protein 1 OS=Homo sapiens OX=9606 GN=STBD1 PE=1 SV=1	1.511	2.082
EHHADH	Q08426	Peroxisomal bifunctional enzyme OS=Homo sapiens OX=9606 GN=EHHADH PE=1 SV=3	1.5085	1.844
